# Supplementary material for: Near infrared fluorescent peptide nanoparticles for enhancing esophageal cancer therapeutic efficacy
Source: Nat Commun. 2018 Jul 4;9:2605. doi: 10.1038/s41467-018-04763-y (PMC6031624; doi:10.1038/s41467-018-04763-y)
Supplement: Supplementary file 1 — Supplementary Information [file 41467_2018_4763_MOESM1_ESM.pdf]

Supplementary Information

Near Infrared Fluorescent Peptide Nanoparticles for Enhancing Esophageal Cancer Therapeutic Efficacy

Zhen Fan[1,2,3,4,5#], Yan Chang[6#], Chaochu Cui[6,7,8#], Leming Sun[9], David H. Wang[10], Zui Pan[6]*, and Mingjun Zhang[1,2,11]*

[1]*Department of Biomedical Engineering, College of Engineering, The Ohio State University, Columbus, OH 43210, USA.*

[2]*Dorothy M. Davis Heart & Lung Research Institute, The Ohio State University Wexner Medical Center, Columbus, OH 43210, USA.*

[3]*Comprehensive Cancer Center, The Ohio State University, Columbus, 43210, USA.*

[4]*School of Material Science and Engineering, Tongji University, Shanghai, 201804, China.*

[5]*Institute for Advanced Study, Tongji University, Shanghai, 200092, China*

[6]*College of Nursing and Health Innovation, The University of Texas at Arlington, Arlington, TX 76019, USA.*

[7]*State Key Laboratory of Oncology in South China; Collaborative Innovation Center for Cancer Medicine, Sun Yat-sen University Cancer Center, Guangzhou, 510060, China.*

[8]*Henan Key Laboratory of Medical Tissue Regeneration, Xinxiang Medical University, Xinxiang, Henan, 453003, China.*

[9]*School of Life Sciences, Northwestern Polytechnical University, Xi'an, 710065, China.*

[10]*Esophageal Diseases Center, Department of Internal Medicine, University of Texas Southwestern Medical Center, Dallas, TX 75390, and VA North Texas Health Care System, Dallas, TX 75216, USA.*

[11]*Interdisciplinary Biophysics Graduate Program, The Ohio State University, Columbus, OH 43210, USA.*

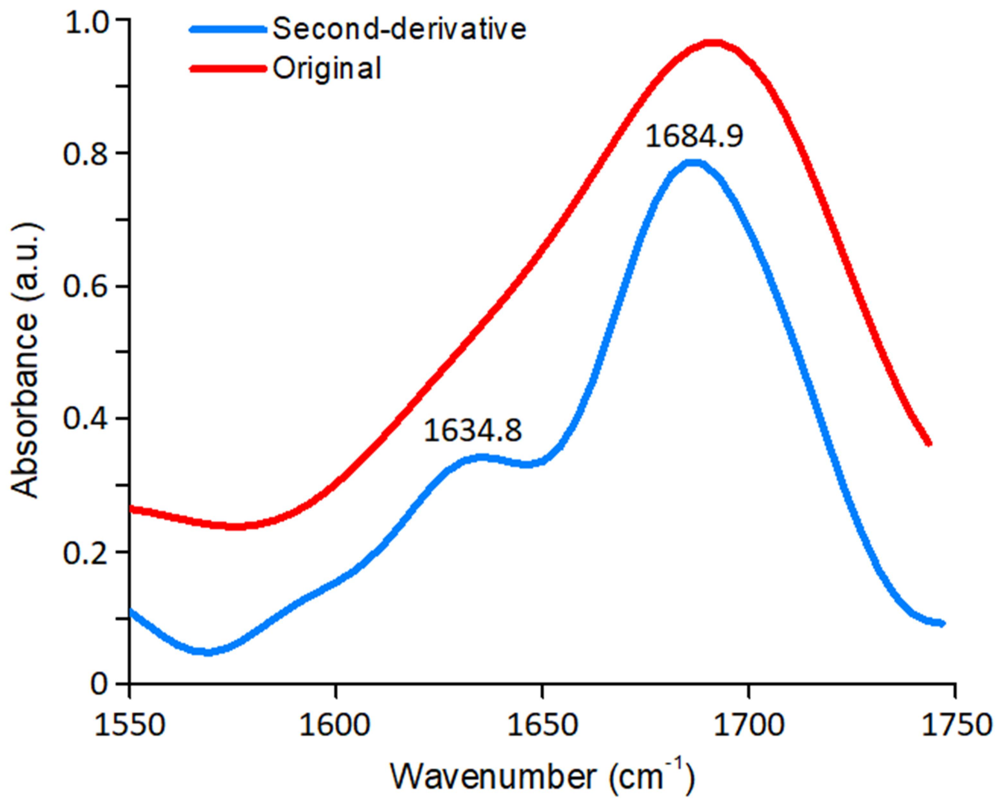

Supplementary Figure 1 FTIR absorbance spectrum of cyclo[-(D-Ala-L-Glu-D-Ala-L-Trp)$_2$-] peptide assemblies. The amide I bands of of 1634.8 and 1684.9 cm^{-1} indicate an antiparallel β-sheet like conformation.

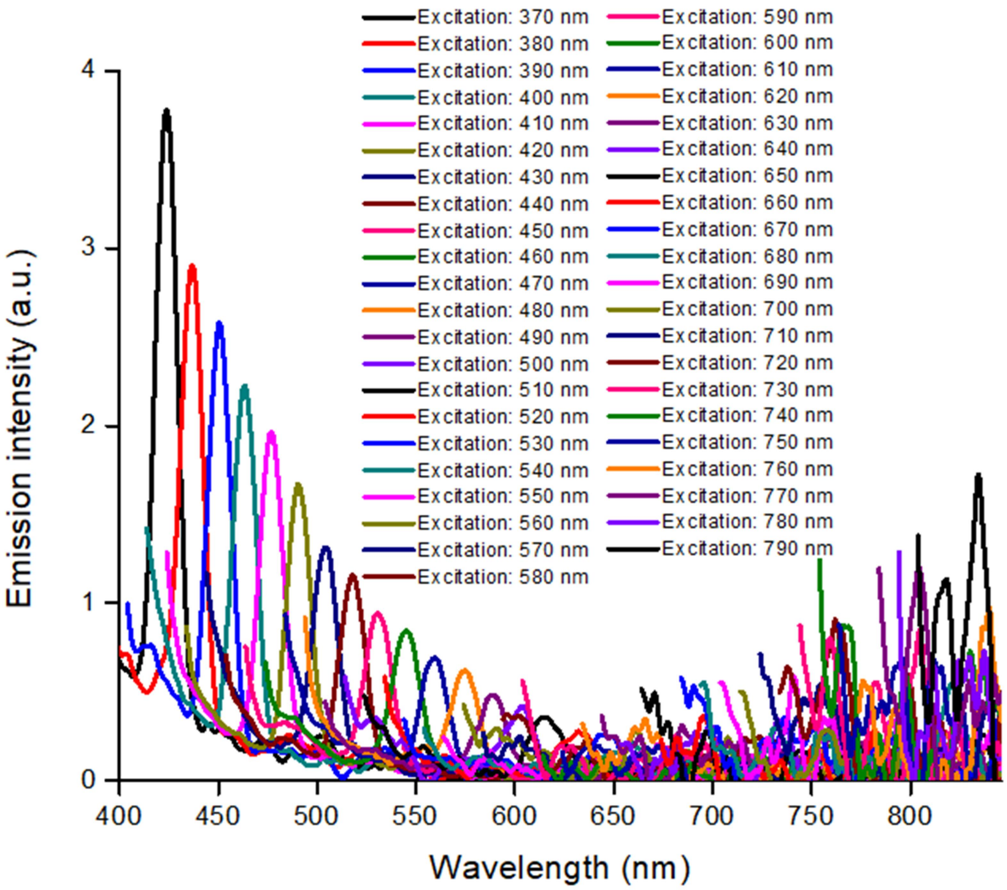

Supplementary Figure 2 Fluorescence emission spectra of pure water with excitation wavelength from 370 to 790 nm intervals of 10 nm.

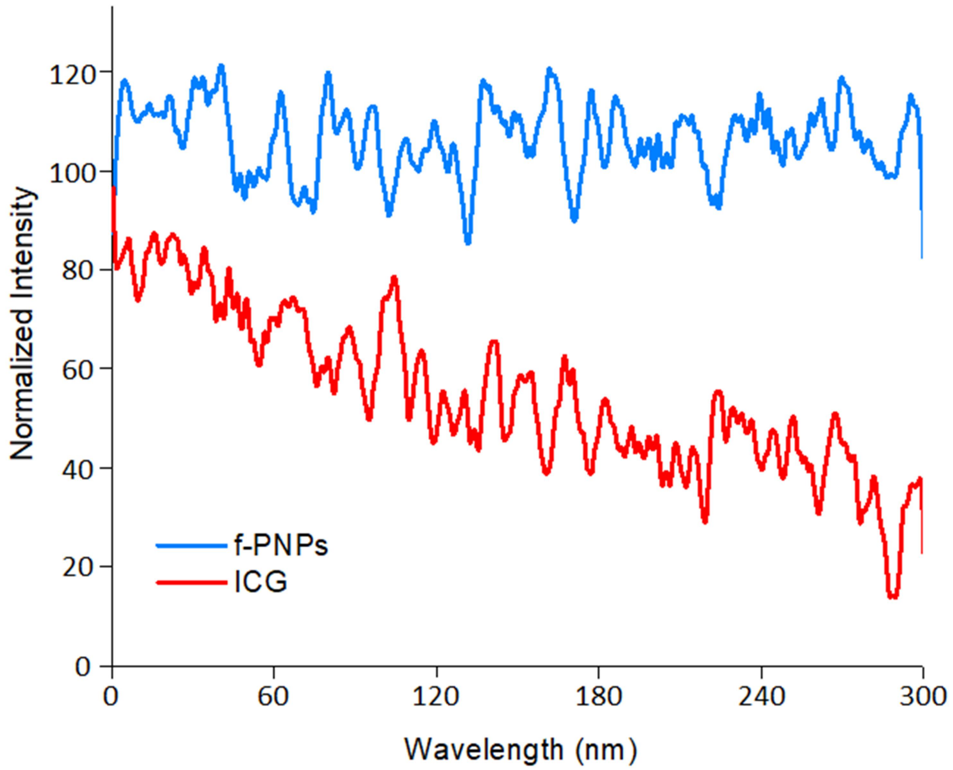

Supplementary Figure 3 Photostability test of the f-PNPs and ICG. The fluorescence intensity of f-PNPs (excitation: 760 nm) remained stable after continuous irradiation for 300 seconds. ICG lost most fluorescence after 300 seconds irradiation.

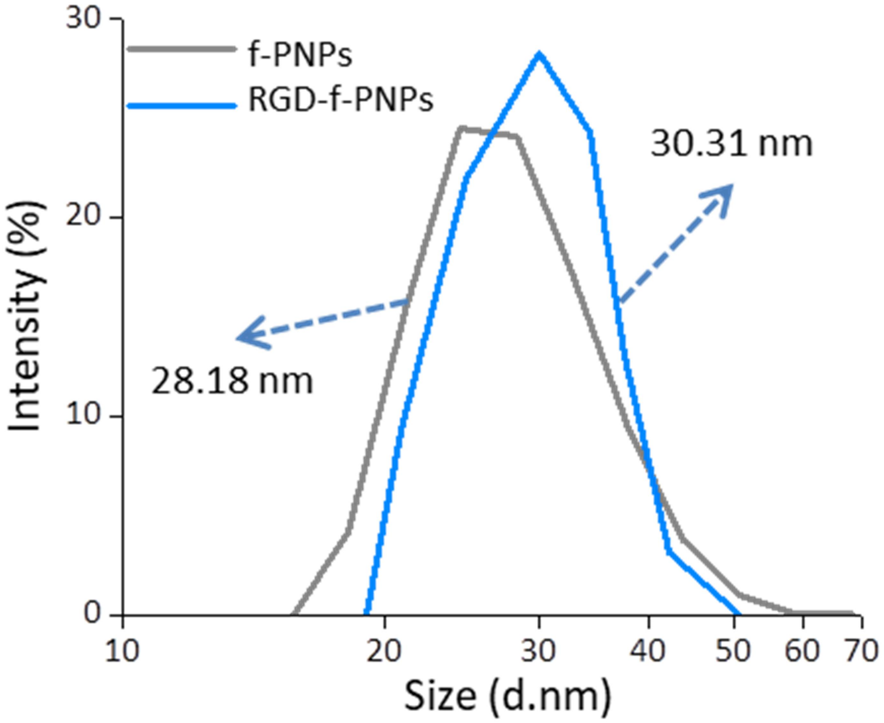

Supplementary Figure 4 Size distribution of the RGD-f-PNPs measured by dynamic light scattering. After the RGD modification, the average diameter of the nanoparticles was slightly increased from 28.18 nm to 30.31 nm.

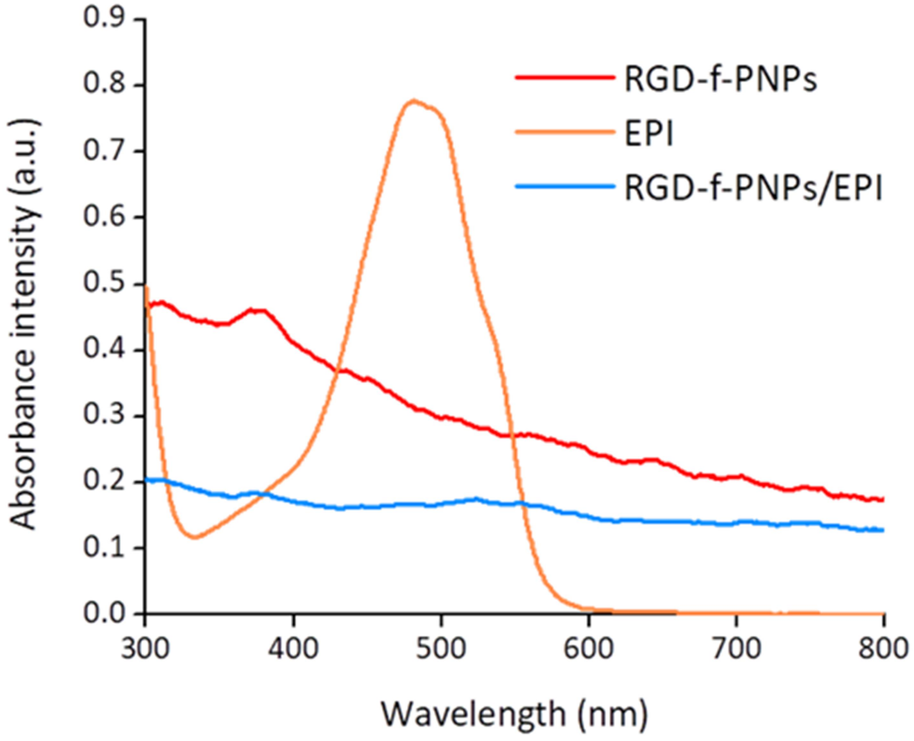

Supplementary Figure 5 Absorbance spectrum of RGD-f-PNPs, EPI and RGD-f-PNPs/EPI conjugates. The absorbance intensity is significantly decreased after conjugation between EPI and RGD-f-PNPs.

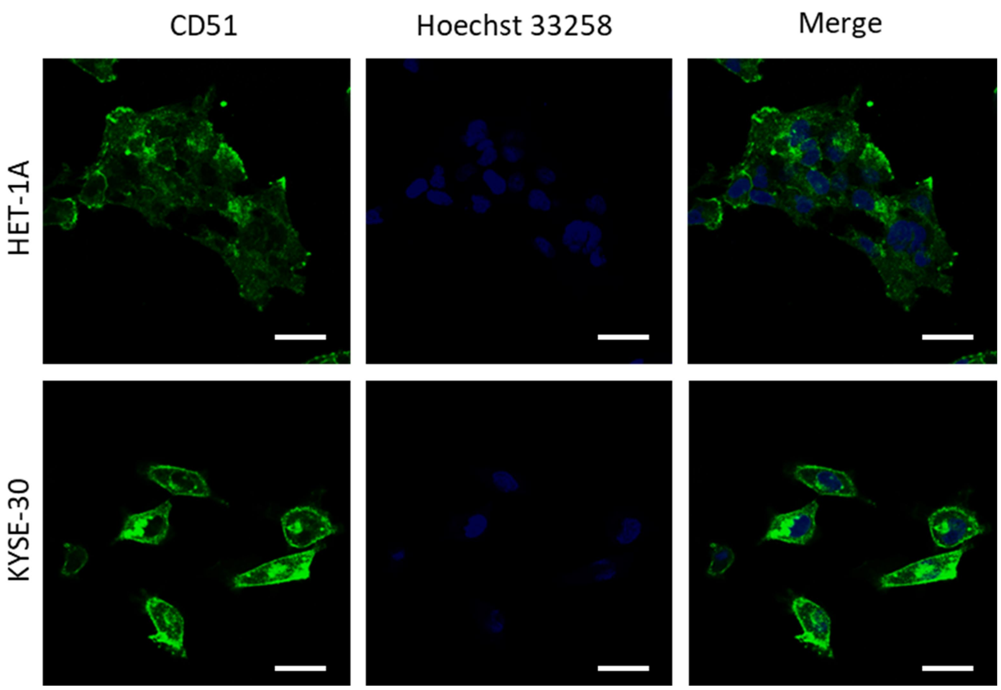

Supplementary Figure 6 CD51 expression detected by confocal microscopy with immunofluorescence staining. Compared to HET-1A cells, KYSE-30 cells showed higher fluorescence intensity at cell surface, which indicated more integrins of KYSE-30 cells. Scale bar 20 μm.

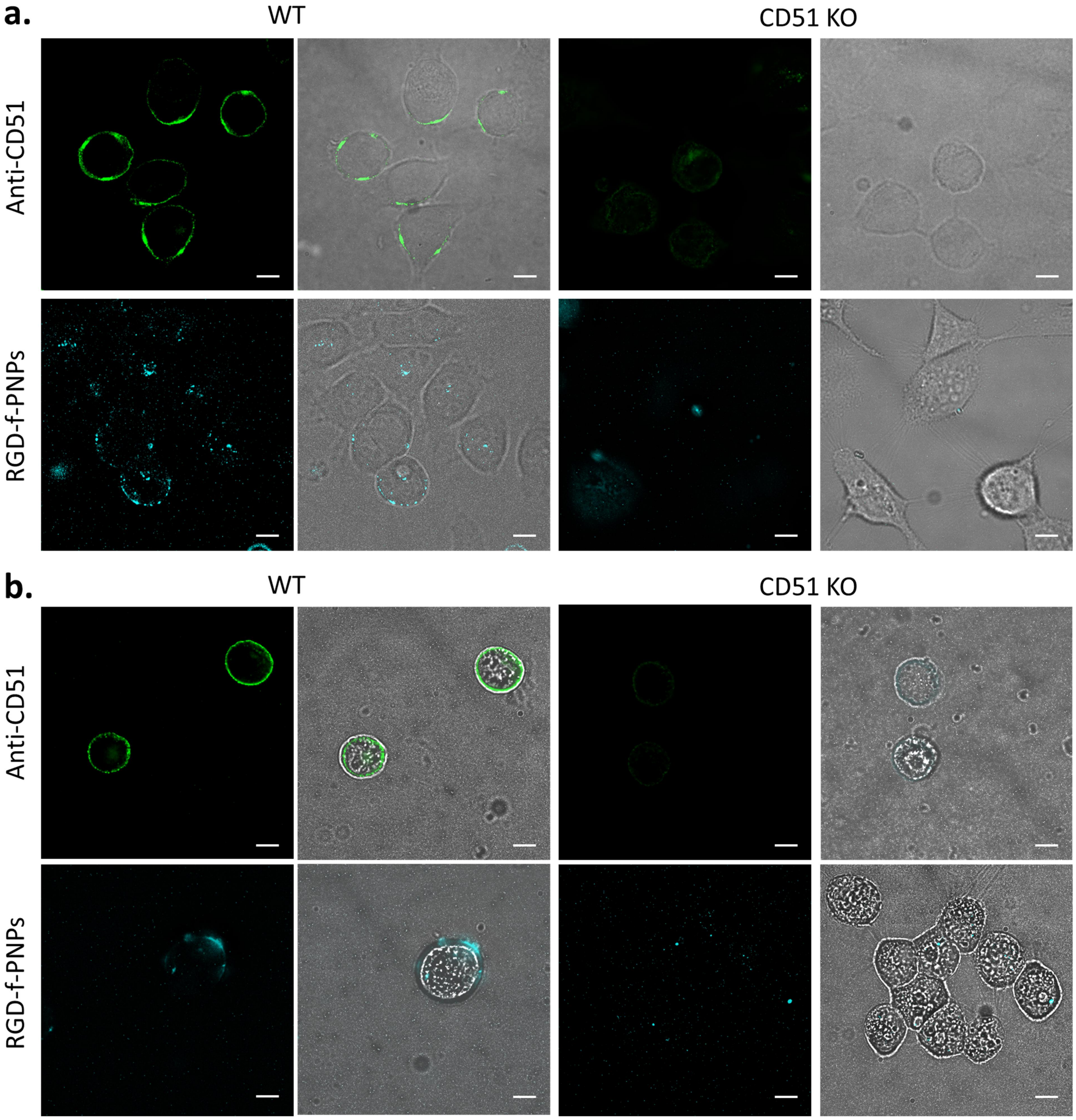

Supplementary Figure 7 CD51 expression and RGD-f-PNPs binding in KYSE-30 and OE33 cells. (a)CD51 expression and RGD-f-PNPs binding in KYSE-30 cells. Cells were incubated with RGD-f-PNPs at 37 °C for 50 minutes. To generate CD51 knockout cells, we inserted gRNAs targeting different regions of gene encoding CD51 into pX330-Cas9 plasmids. The plasmids were co-transfected into KYSE-30 cells. Scale bar 10 µm. (b) CD51 expression and

RGD-f-PNPs binding in OE33 cells. Cells were incubated with RGD-f-PNPs at 37 °C for 50 minutes. To generate CD51 knockout cells, we inserted gRNAs targeting different regions of gene encoding CD51 into pX330-Cas9 plasmids. The plasmids were co-transfected into OE33 cells. Scale bar 10 μm. WT, wild-type cell. KO, knockout cell.

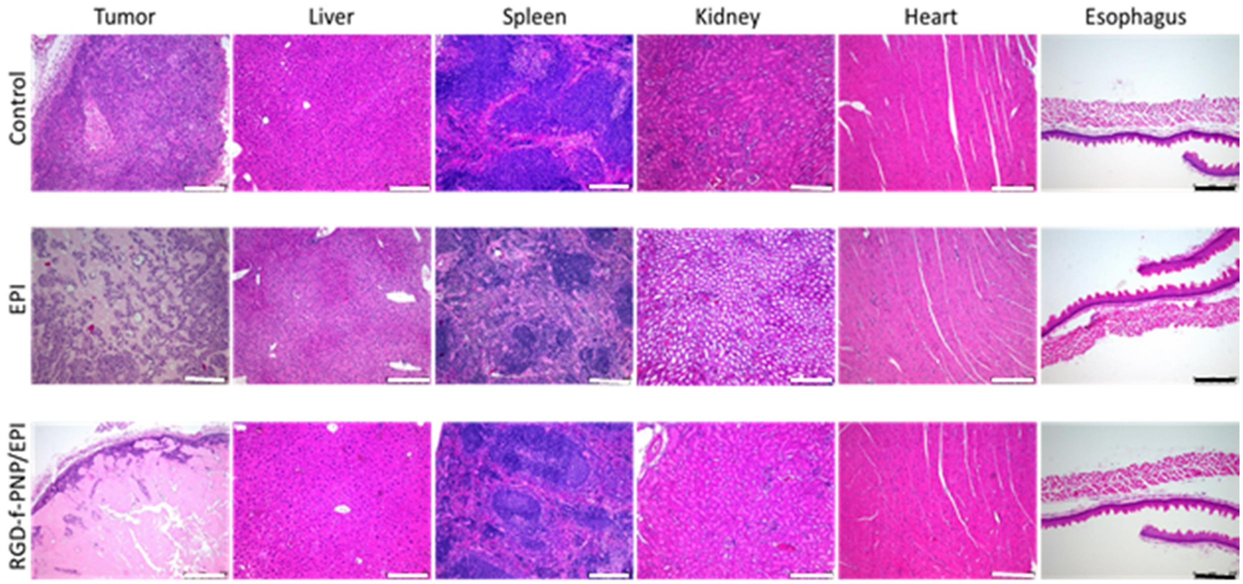

Supplementary Figure 8 Hematoxylin and eosin (H&E) staining was performed on postmortem tissues to examine the damage in the hearts, livers, kidneys and spleens removed from the 4 groups of animals. Normal morphology was observed in the spleens from all four groups, indicating no obvious toxicity to spleen and no severe immune cell cytotoxicity in all groups. EPI at 6 mg/kg caused significant body weight lost and histological changes in kidney, liver and heart, especially. In the hearts, cardiomyocytes demonstrated significant swelling with associated, degenerative and necrotic areas. Compared with EPI group, RGD-f-PNPs/EPI treated animals showed essentially normal kidney and liver morphology, fewer and smaller necrotic areas in heart, indicating that RGD-f-PNPs/EPI caused no or much less damage in these organs. Scale bar: 250 μm.

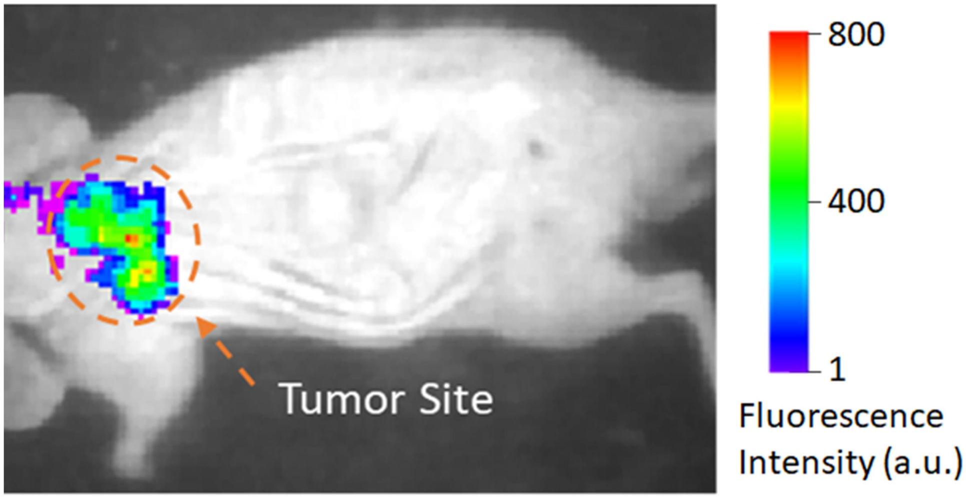

Supplementary Figure 9 Representative *in vivo* imaging of NIR fluorescence after intravenous injection of RGD-f-PNPs *via* the tail vein. The animals were fed with Alfalfa-free diet (Envigo) to eliminate the autofluorescence from regular chow. 6 hours of injection, the xenograft tumors were clearly visualized in live animal NIR imaging at relative low background. The excitation and emission wavelengths are 690 nm and 830 nm, respectively.
